# A graph-based algorithm for estimating clonal haplotypes of tumor sample from sequencing data

**DOI:** 10.1186/s12920-018-0457-4

**Published:** 2019-01-31

**Authors:** Yixuan Wang, Xuanping Zhang, Shuai Ding, Yu Geng, Jianye Liu, Zhongmeng Zhao, Rong Zhang, Xiao Xiao, Jiayin Wang

**Affiliations:** 1 Department of Computer Science and Technology, School of Electronic and Information Engineering, Xi’an Jiaotong University, Xi’an, 710048 China; 2 Shaanxi Engineering Research Center of Medical and Health Big Data, School of Electronic and Information Engineering, Xi’an Jiaotong University, Xi’an, 710048 China; 3 School of Management, Ministry of Education Key Laboratory of Process Optimization and Intelligent Decision-Making, Hefei University of Technology, Hefei, 23009 China; 4 Institute of Health Administration and Policy, School of Public Policy and Administration, Xi’an Jiaotong University, Xi’an, 710048 China

**Keywords:** Cancer genomics, Haplotype phasing, Clonal haplotype, Computational pipeline, Sequencing data analysis

## Abstract

**Background:**

Haplotype phasing is an important step in many bioinformatics workflows. In cancer genomics, it is suggested that reconstructing the clonal haplotypes of a tumor sample could facilitate a comprehensive understanding of its clonal architecture and further provide valuable reference in clinical diagnosis and treatment. However, the sequencing data is an admixture of reads sampled from different clonal haplotypes, which complicates the computational problem by exponentially increasing the solution-space and leads the existing algorithms to an unacceptable time-/space- complexity. In addition, the evolutionary process among clonal haplotypes further weakens those algorithms by bringing indistinguishable candidate solutions.

**Results:**

To improve the algorithmic performance of phasing clonal haplotypes, in this article, we propose *MixSubHap*, which is a graph-based computational pipeline working on cancer sequencing data. To reduce the computation complexity, *MixSubHap* adopts three bounding strategies to limit the solution space and filter out false positive candidates. It first estimates the global clonal structure by clustering the variant allelic frequencies on sampled point mutations. This offers a priori on the number of clonal haplotypes when copy-number variations are not considered. Then, it utilizes a greedy extension algorithm to approximately find the longest linkage of the locally assembled contigs. Finally, it incorporates a read-depth stripping algorithm to filter out false linkages according to the posterior estimation of tumor purity and the estimated percentage of each sub-clone in the sample. A series of experiments are conducted to verify the performance of the proposed pipeline.

**Conclusions:**

The results demonstrate that *MixSubHap* is able to identify about 90% on average of the preset clonal haplotypes under different simulation configurations. Especially, *MixSubHap* is robust when decreasing the mutation rates, in which cases the longest assembled contig could reach to 10kbps, while the accuracy of assigning a mutation to its haplotype still keeps more than 60% on average. *MixSubHap* is considered as a practical algorithm to reconstruct clonal haplotypes from cancer sequencing data. The source codes have been uploaded and maintained at https://github.com/YixuanWang1120/MixSubHap for academic use only.

## Background

Modern canceration theory summarizes that tumor heterogeneity is one of the key results of tumor proliferation and evolution [1]. Any macroscopic tumor tissue is considered as an admixture of cancerous and non-cancerous cells, where the cancerous cells, in many cases, could be further clustered into multiple sub-clones, according to their somatic mutational events [2, 3]. These somatic mutations, interacting with germline variations, often underlie different deleterious selective advantages, which may further contribute to drug resistance, tumor recurrence and metastasis, and many other phenotypes [4–6]. For example, it is observed that the presence of multiple sub-clones could be associated with poor clinical outcomes in a group of chronic lymphocytic leukemia cases [7]. It is also reported that the clonal competition for predominance occurs spontaneously in multiple myeloma cases and the heterogeneous clonal mixtures may shift predominant clones with therapeutic selection [8]. Not only in blood cancer cases, similar conclusions could be drawn in many other cancer types, such as esophageal adenocarcinoma [9], lung adenocarcinoma [10] and renal clear cell carcinoma [11]. It is now a popular opinion that a comprehensive understanding on tumor heterogeneity benefits clinical diagnosis and potential precision treatments.

Genomic analysis on tumor heterogeneity has two levels: genotype level and haplotype level. The genotype-level bioinformatics pipelines differentiate homozygous mutational events, including loss of heterozygosity (LOH), from heterozygous ones [12–14], and then cluster them into sub-clones [3, 15]. On this basis, the haplotype level analysis requires locating each heterozygous mutation on the corresponding chromosomal sequence of alleles, named haplotype, whose computational problem is often called *haplotype phasing*. Actually, haplotype phasing has already been an important step in many bioinformatics workflows besides cancer research [16], but its importance in cancer genomics is recently emphasized [17]. Understanding haplotype heterogeneity is suggested not only to elucidate a series of critical genome-to-transcriptome events, e.g. gene fusion transcripts and their driver partners [18], but to facilitate the studies on the interactions among different germline and somatic variations, e.g. two-hit events and allelic amplifications [4, 5, 17]. Such results could significantly benefit downstream analyses and studies in many fields, including disease association studies [6, 19], clinical decision-support with electronic medical record data [20–22], drug and treatment designs and improvements [23, 24], etc.

Benefiting from the second generation sequencing technology, tens of thousands of cancer patients have been sequenced, and the cancer sequencing data have been accumulating rapidly as well, which greatly promotes the studies on clonal heterogeneity and expansion [25] and the developments of related computational approaches [26]. Nowadays, tumor heterogeneity analyses are almost built up on cancer sequencing data. Although the existing approaches differ in models, algorithms, and the evaluation standards for “well suited”, based on our best knowledge, the core algorithms may be roughly divided into two categories: the phylogenetic model-based methods and model-free ones. The phylogenetic model-based methods, just as the name implies, usually focus on the computational problem of inferring phylogenetic trees that describe clonal expansion and evolution [27–32]: *TrAp* proposed an expanded algorithm from a brute-force algorithm for sub-clonal deconvolution, which generated the evolutionary tree(s) by comprising the maximum number of first-generation trees [27]. *PhyloWGS* established a probabilistic model for phylogeny inference, which incorporated the information of variant allele frequencies and the estimations of allelic amplifications and LOHs [28]. *BitPhylogeny* designed a graphical model, and then it adopted two strategies, which were a Markov chain Monte Carlo (MCMC) sampling and a maximum posterior method on expected adjusted rand, to solve the possible phylogenies [29]. *SPRUCE* utilized a bounded enumeration strategy to search the solution space of candidate perfect phylogenies which were consistent with the given data set [30]. *Canopy* improved the statistical framework and was capable of handling the data sequenced from temporally and/or spatially separated samples from the same patient to reconstruct tumor phylogeny [31]. A recent published method further addressed the lack of methods for tumor deconvolution and phylogenetics of diverse classes of structural variations at base-pair resolution [32].

On the other hand, it is argued that the specific features of tumor evolution may challenge the direct applications of classical phylogenetic models [33]. One of the key issues occurs when classical phylogenetic approaches require a priori on the number of sub-clones, which is an unknown parameter for cancer sequencing data. To overcome such issues, the model-free methods often focus on the clonal structures with the maximum likelihood on global variant allelic frequencies [3, 34–38]: *THetA* designed a convex optimization algorithm to solve the maximum likelihood mixture decomposition, which optimized the multinomial probability [34]. *PhyloSub* proposed a series of topological constraint rules to limit the possible phylogenies that were able to explain the frequency changes [35]. *PyClone* introduced a Bayesian clustering method, which integrated the estimations on cellular prevalences, normal-cell contamination and segmental copy-number changes [36]. *SciClone* adopted a variational Bayesian mixture model to provide a global estimation of clonal architecture across all of the given copy-number neutral regions [3]. *TITAN* established a graphical model to estimate sub-populations based on copy number alterations and loss of heterozygosity events [37]. Automate learning was also incorporated for deconvolution of genomic mixtures, where the RNA expression data was involved in addition to improve the performance [38]. In general, there is no clear boundary between the two categories, and several comprehensive reviews compared the advantages among the existing approaches [26, 33].

However, most of the existing approaches are not able to deepen the analyses to haplotype level efficiently. When multiple haplotypes are considered, the evolutionary process should be represented by a set of parallel phylogenetic trees rather than possible single phylogenies, which is different from the hypothesis on which most of the existing methods, the phylogenetic model-based methods or model-free ones, rely [3, 28–30, 32, 34, 36–38]. For those methods considering concurrent evolutionary processes, haplotype phasing algorithms are needed to locate heterozygous mutations prior to inferring clonal structure [27, 31, 35]. Moreover, phasing multiple haplotypes is also a quite challenging computational problem because the solution space of possible haplotypes is exponentially increased along with the increasing of sub-clones. For example, for *k* sub-clones each with *n* heterozygous variation sites, the solution space of 2*k* clonal haplotypes (allelic imbalance events are not considered) reaches *O*(2^(2*k*−1)*n*^) [39]. The polyploid phasing problem has already been suggested as an NP-hard problem [39–44], hence probabilistic algorithms and heuristic strategies are commonly used to approximate optimization solutions, such as Gibbs sampling [39], greedy binning algorithm [41], branch-and-bound scheme by maximum likelihoods [42], semi-definite programming [43], sparse tensor decomposition [44], etc.

Different from polyploid haplotypes, the haplotypes of multiple sub-clones from the same sample always imply the information of its clonal structure. Thus, to enhance the efficiency of the existing computational pipeline, e.g. [17], we consider to incorporate the priori of clonal structure to bound the solution space, and then polish the clonal structure with estimated clonal haplotypes. To achieve this, in this article, we propose *MixSubHap*, a computational pipeline for phasing clonal haplotypes as well as estimating clonal structure. To reduce the computation complexity, *MixSubHap* adopts three bounding strategies to limit the solution space and filter out false positive candidates. It first estimates the global clonal structure by clustering the variant allelic frequencies on sampled point mutations. This offers a priori on the number of clonal haplotypes when copy-number variations are not considered. Then, it utilizes a greedy extension algorithm to approximately find the longest linkage of the locally assembled contigs. Finally, it incorporates a read-depth stripping algorithm to filter out false linkages according to the posterior estimation of tumor purity and the estimated percentage of each sub-clone in the sample.

## Methods

**Fig. 1:**
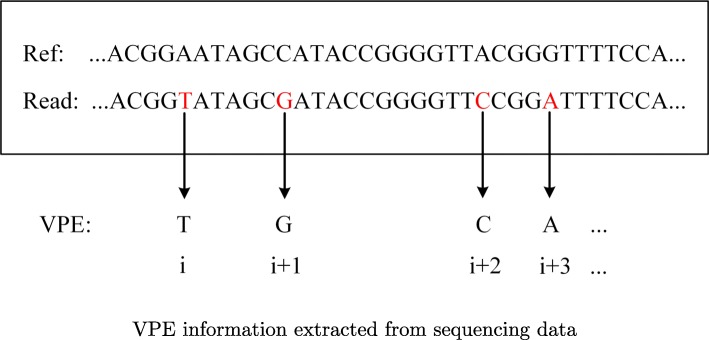
Extracting a VPE from a pair of mapped reads. When a read-pair is mapped to the reference genome, the corresponding VPE consists of the sites with variants

Suppose that we are given a set of paired-end sequencing data with mapping information, and the outputs of the proposed pipeline include both the number of sub-clones and the haplotypes of each sub-clone. The given data is first pre-processed: a read is retained if it brings at least one point mutations, while a read-pair is retained if it brings at least two point mutations. Each read-pair that passed the filter is then collapsed to a much shorter sequence, named VPE as in [45], by extracting the sites with point mutations from this read-pair, as shown in Fig. 1. A VPE consists of only the sites with variants from the corresponding read-pair. In the current stage of this research, the structural variations, including the allelic imbalance events on point mutations, are temporarily not considered. According to these reads (VPEs), the variant allelic frequency (VAF) of each variant is calculated. We adopt a model-free method to provide a priori of the clonal structure according to global variant allelic frequencies. A series of model-free method could achieve this. Here, we incorporate *SciClone* [3], a popular method, into the proposed pipeline.

**Fig. 2:**
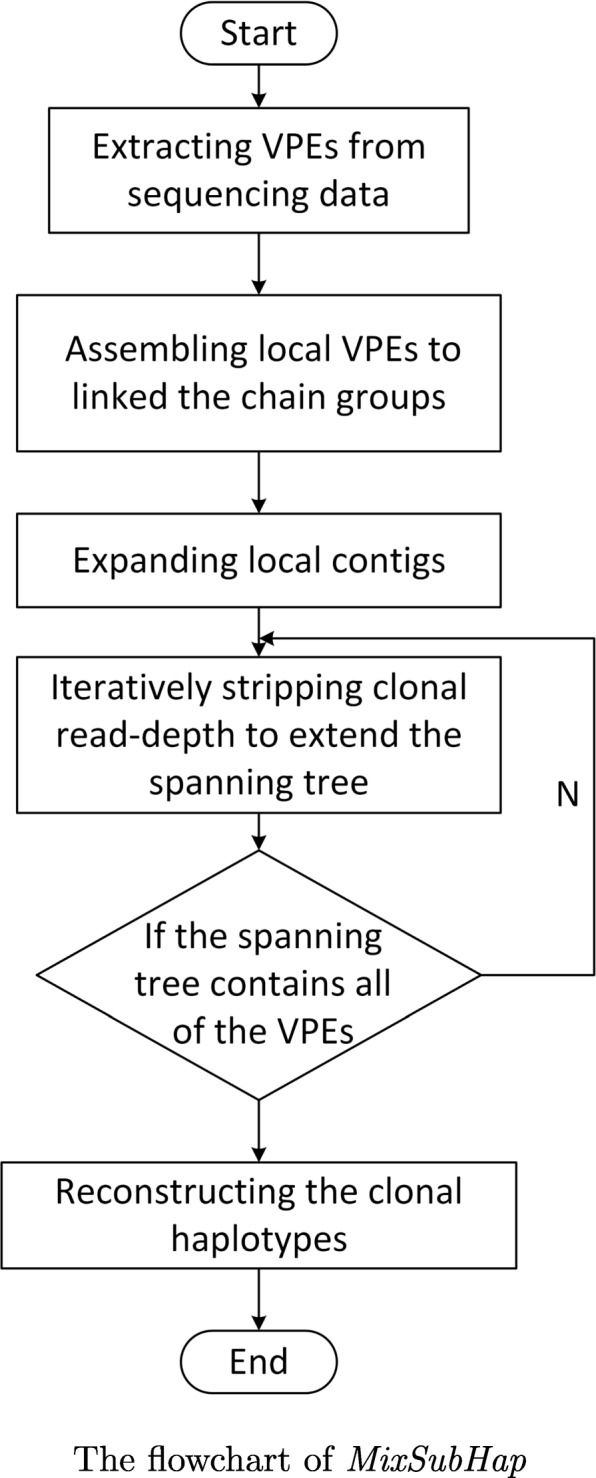
The flowchart of the proposed pipeline

*MixSubHap* consists of three major components: assembling local VPEs, expanding local contigs and iteratively stripping clonal read-depth. The flowchart is shown in Fig. 2.

- Assembling local VPEs: As the first component, a divide-and-conquer strategy is adopted to assemble the VPEs to multiple groups of contigs. Different from assembling reads, VPEs are first clustered by starting sites, and then for each cluster, the VPEs with the same starting site are processed together to form a small set of contigs. Note that, multiple reads whose mapping positions are slightly different may collapse to the same VPE sequence if they bring the same variants. In addition, several constraints which imply the inheritance principle across sub-clones are applied. The details of these constraints are further discussed in Discussion section.
- Expanding local contigs: To link the contigs across different clusters, an efficient greedy algorithm is conducted. An undirected weighted graph is established. Each vertex represents a variant site. An edge exists between two vertexes if at least one VPE supports the linkage of them. The edges are weighted according to the likelihoods of possible linkage modes. The greedy algorithm first generates a maximum spanning tree on the sub-graph that consists of the vertexes from the founding clone. Here, the estimation of founding clone is provided by *SciClone*. Moreover, the computation is also simplified by the inheritance constraint, which limits the linkage mode in all of the descendant sub-clones.
- Stripping each clonal read-depth: For the variants do not occur in the founding clone, different genotypes are brought by different sub-clones. In these cases, the likelihoods are interfered by allelic clonal haplotypes. To filter out the bias on the likelihoods, a thickness stripping algorithm is designed. According to the VAFs and the estimations on clonal structure, the VPEs that have higher probabilities of sampling from other sub-clones are removed, and then the spanning tree is extended based on the corrected edge weights. The parental haplotypes guide the clonal haplotypes of its descendants. When the tree is traversed, the clonal haplotypes are then reconstructed.

### Assembling local VPEs

Let **V** denote the set of given variant sites. VPEs are defined as the information which contain the base states of all the mutated sites along with their actual positions in reads and relative positions in set **V**. When extracting VPE information, it is necessary to ensure that the number of mutations brought by the read or read-pair is not less than two. Sequencing reads that contain only one mutation do not reflect the interrelationship between mutations and do not provide useful information for haplotype reconstruction, thus we do not take such information into consideration. It is assumed that each sequence library is well prepared and the insert-size obeys a normal distribution with a small variance. Therefore, libraries with different lengths are introduced, more variant sites can be extracted correctly.

In general, the rare somatic mutations on cancer susceptibility genes may reach 10,000, which is a huge challenge for the computational capability of ordinary computer. At this time, the computational complexity is too high due to the large amount of variants. Therefore, the *MixSubHap* uses the Dividing and Assembling strategy to process the VPE information. The strategy appropriately clusters the length of the division and designs the connection ways, so that the resulting short chains can accurately exhibit fragments of the sub-clonal haplotypes. To achieve this, the VPE is first mapped to the reference by mapping the base state of corresponding variant against the reference according to its actual position. After that, the aligned VPE are divided into *M* groups according to its starting position, then the algorithm processes group by group. Assume that the number of sub-clones *I* is known, then the number of haplotypes is determined, which is 2*I*, and the total number of variants is *N*. The process consists of the following steps:

- Sorting the VPEs via the starting positions.
- Constructing initial short chain groups. VPE alignments at the same starting position are processed together to form a group of short chains. Specifically, this is an integer programming problem, where the goal of the programming is that the cardinality of a set of short chains (the number of short chains in the group) is minimal, and the constraint of the programming is that the short-chain group must be able to support all the corresponding VPEs, carrying the maximum number of variant sites. During solving this, the greedy strategy is used to minimize the number of short chains, and at the same time to support all the VPE information of this group. In order to ensure that the ambiguity chain does not produce redundant, we add each VPE into a short chain group only when the VPE not contained in the existing short chains, otherwise, the VPE remains in the candidate set and the short chain group retains the same.
- According to the principle of inheritance, arrange all short chain groups and keep qualified arrangement. Process VPEs whose starting sites are *p* (*p*∈*N*), then process group by group, until all VPEs are processed.

Until now, all the VPEs are rationally and effectively connected, and a large number of short chain groups carrying haplotype information are formed. Next, we will present a reasonable and efficient strategy to further construct the haplotypes of each sub-clone.

### Expanding local contigs

According to the hypothesis of linear evolution mode between tumor sub-clones (see in Discussion), we know that, once the connection mode of a pair of variants from the founding clone *S*_0_ is fixed in the tumor evolutionary process, the differentiation of subsequent sub-clone *S*_*i*_, *i*∈{1,2,…,*I*−1} will inherit the same connection without any change. In the same way, once the connection between a pair of variants from sub-clone *S*_*i*_ at any level is confirmed, it will not change in its descendant clone *S*_*j*_(*i*<*j*). According to the linear evolution mode, the mutation sites in the clones are separated layer by layer. Thus, the parental clonal haplotype structure can be used as a known condition to guide the construction of the descendant clonal haplotypes.

In order to recognize the clonal haplotypes efficiently and accurately, *MixSubHap* algorithm first clusters all the variant sites according to the VAF of each site. The variants in the same cluster are from the same sub-clone [3], and then the clustering results are considered as the basis to initialize the clonal haplotypes. The clustering method used in this paper is *SciClone* version 1.1.0 [3], which is reported to be relatively accurate in clustering the somatic mutations by clonal structure.

*MixSubHap* algorithm mainly generates a maximum spanning tree based on the short chain groups we obtained. Let *M* be the set of all variants, and 〈*p*_*i*_,*p*_*j*_〉 represent two adjacent allelic sites, *p*_*i*_,*p*_*j*_∈*M*. $ \text {Let} \, H^{p_{i},p_{j}}_{S_{k}} $ represent the connection mode between the *p*_*i*_th variant site and the *p*_*j*_th variant site from the sub-clone *S*_*k*_. *A* stands for the same base as the reference genome, while *B* stands for a base different from the reference genome, namely *B* stands for a mutation. Therefore, for any two sites from the same sub-clone, possible values for $ H^{p_{i},p_{j}}_{S_{k}} $ are {(*A*,*A*), (*B*,*B*)}*or*{(*A*,*B*), (*B*,*A*)}. For the founding clone *S*_0_, according to the short chain groups, we can get many different values of $ H^{p_{i},p_{j}}_{S_{0}} $ and add these different values respectively to the corresponding coverage of variant pair 〈*p*_*i*_,*p*_*j*_〉. In order to separate the sub-clones layer by layer, variant pair 〈*p*_*i*_,*p*_*j*_〉 from every sub-clone and the corresponding coverage level *c*_〈*i*,*j*〉_ are used to estimate the clonal haplotypes. For variant pairs, the coverage of each pair and the probability of various possible connection modes between the two variants are calculated together. According to the probabilities of various connection patterns, the corresponding undirected weighted graphs are established. When calculating the coverage level *c*_〈*i*,*j*〉_ of allelic sites 〈*p*_*i*_,*p*_*j*_〉, only short chain groups are considered. Let *F*(*i*,*j*) represent the set of short chains containing both allelic sites 〈*p*_*i*_,*p*_*j*_〉. |*F*(*i*,*j*)| represents the number of chains in this set. We set the connection probability of {(*A*,*A*), (*B*,*B*)} to be positive and set the connection probability of {(*A*,*B*), (*B*,*A*)} to be negative. Let *G* represent a weighted undirected graph where the variant sites are the vertexes of the graph. Here, we set the coverage threshold *Cov*, with a default value of 2. If |*F*(*i*,*j*)|≥*Cov*, there will be an edge between variants 〈*p*_*i*_,*p*_*j*_〉 and the weight of edge will be the probability of the connection. The formula of weight is:

$$W_{\left\langle i,j\right\rangle} = f\left(b_{p_{i}},b_{p_{j}},A\right)- f\left(b_{p_{i}},b_{p_{j}},B\right) $$

$$N^{b_{p_{i}},b_{p_{j}}}(p_{i},p_{j}) = \sum_{r \in F(i,j)}I\left(r(p_{i},p_{j})=\left(b_{p_{i}},b_{p_{j}}\right)\right) $$

Where, $ N^{b_{p_{i}},b_{p_{j}}}(p_{i},p_{j}) $ represents the number of chains across allelic sites 〈*p*_*i*_,*p*_*j*_〉 corresponding to the connection mode $ (b_{p_{i}},b_{p_{j}}) $. $b_{p_{i}} $ and $ b_{p_{j}} $ respectively indicate the base states of the variant site *p*_*i*_ and *p*_*j*_. *r*(*p*_*i*_,*p*_*j*_) represents alleles for variant site *p*_*i*_ and *p*_*j*_. There are four kinds of joint states for allele *p*_*i*_ and *p*_*j*_, $ \text {where} \; (b_{p_{i}},b_{p_{j}}) \in \left \lbrace (A, B), (B, A), (A, A), (B, B) \right \rbrace $. *I*(.) is the indicator function. When $\phantom {\dot {i}\!} r(p_{i}, p_{j}) = (b_{p_{i}},b_{p_{j}}) $ is true, the function value is 1, 0 otherwise. For variant site *p*_*i*_, consider the sequencing error and alignment error of each site *ε*.

Let

$$N^{A}(p_{i},p_{j}) = N^{A,A}(p_{i},p_{j})+N^{B,B}(p_{i},p_{j}) $$

$$N^{B}(p_{i},p_{j}) = N^{A,B}(p_{i},p_{j})+N^{B,A}(p_{i},p_{j}) $$

Two connection probabilities of the paired variant sites 〈*p*_*i*_,*p*_*j*_〉 are,

$$ {\begin{aligned} f(b_{p_{i}},b_{p_{j}},A) = \frac{ \left((1-\epsilon)^{2} + \epsilon^{2}\right) \times N^{A}(p_{i},p_{j}) + 2\epsilon \times (1-\epsilon) \times N^{B}(p_{i},p_{j}) }{ \left| F(i,j) \right| } \end{aligned}} $$

$$ {\begin{aligned} f(b_{p_{i}},b_{p_{j}},B)=\frac{ \left((1-\epsilon)^{2} + \epsilon^{2}\right) \times N^{B}(p_{i},p_{j}) + 2\epsilon \times (1-\epsilon) \times N^{A}(p_{i},p_{j}) }{ \left| F(i,j) \right| } \end{aligned}}  $$

Where *W*_〈*i*,*j*〉_>0 indicates that the connection mode of allelic sites 〈*p*_*i*_,*p*_*j*_〉 is $ H^{A,A}_{S_{0}}/ H^{B,B}_{S_{0}}$; otherwise, *W*_〈*i*,*j*〉_<0 indicates that the connection mode of allelic sites 〈*p*_*i*_,*p*_*j*_〉 is $ H^{A,B}_{S_{0}}/ H^{B,A}_{S_{0}}$. The greater the absolute value of *W*_〈*i*,*j*〉_, the higher the reliability of the corresponding connection pattern.

**Fig. 3:**
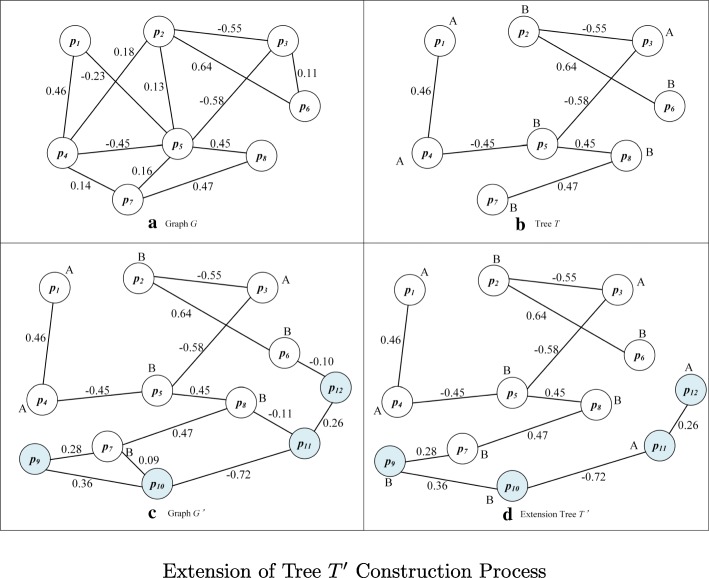
Extension of Tree *T*^′^ Construction Process

After constructing the undirected weighted graph *G* of the founding clone is constructed, the number of vertexes in the graph is *N*_*v*_. All the vertexes in the graph *G* are all derived from variant sites in the founding clone *S*_0_. The algorithm selects a vertex, whose base state is known, as the starting point *sp* for constructing the sub-clonal haplotypes and generating the initial maximum spanning tree *T* corresponding to graph *G*. The processing steps are as follows, as shown in Fig. 3:

- Find all variant sites connected to *sp* from graph *G*;
- Select the corresponding edge of *max*(|*W*_〈*i*,*j*〉_|) as one edge of the maximum spanning tree *T*;
- According to the positive or negative of the edge weight, the state of another variant connected to *sp* can be determined and these variants will be considered as new known vertexes;
- Conduct the second step successively for other unkonwn variants and repeat adding border process to maximum spanning tree *T* till all the variants have appeared in *T* or cannot be added any more.

In actual, due to the sparseness of the founding clone variants and the limitation of the read length of the second generation sequencing data, the generated undirected weighted graph *G* is often not a connected graph, but consists of several mutually disconnected subgraphs. *MixSubHap* algorithm generates an equal number of subtrees on these subgraphs and identifies the base states, thus guides the later extension of the spanning tree.

### Stripping each clonal read-depth

After the initial maximum spanning tree *T* is built, the connection patterns of partial variant sites on the haplotypes have been determined. However, there are a large quantity of variant sites not included in the initial maximum spanning tree *T*. The connection modes of these variant sites are relatively complex, including three types of linkages (*B*,*A*),(*A*,*A*),(*A*,*B*) with varying proportion of each according to the sub-clone proportion in the tumor sample. So we adopt the thickness stripping strategy to strip the read depth level by level from the founding clone to the uppermost layer descendant sub-clone. The remaining sub-clones can be processed in accordance with the method of constructing tree in the founding clone.

Thickness stripping strategy refers to finding the separation point that identifies the current sub-clone and divides all the clones into two parts in the direction of alleles. If sub-clone *S*_*i*_ is the first sub-clone which two variants have been mutated, this sub-clone should be considered as the demarcation line and the upper parental clone of this sub-clone should be separated at a mixed ratio. With the process of evolution, the variant allelic frequency is decreasing, so the initial value of sub-clone proportion should be estimated according to the mean of allelic frequency of each sub-clone. After separation, there are only two types of connection ways between allelic sites for remaining sub-clones and the weights of the two ways are the same in positive and negative. So the connection between the allelic sites can be clearly judged. For the portion of sub-clones to be stripped, they can be separated from the mixed sequencing data according to the mixed ratio of the sub-clones. Set the number of clones to be *N*_*s*_, and then the thickness stripping formula is:

$$P^{A}(p_{i},p_{j}) = \left| F(p_{i},p_{j})\right| \times \left(\sum\limits_{0 \leq i \leq S_{k}} r_{i} + \sum\limits_{S_{k+1} \leq i \leq S_{N_{s}}} \frac{1}{2} r_{i}\right) $$

$$P^{B}(p_{i},p_{j}) = \left| F(p_{i},p_{j})\right| \times \left(\sum\limits_{S_{k+1} \leq i \leq S_{N_{s}}} \frac{1}{2} r_{i}\right) $$

Adjust the coverage of the allelic site 〈*p*_*i*_,*p*_*j*_〉, we have

$$\hat{N^{A}}(p_{i},p_{j}) = N^{A}(p_{i},p_{j}) - P^{A}(p_{i},p_{j}) $$

$$\hat{N^{B}}(p_{i},p_{j}) = N^{B}(p_{i},p_{j}) - P^{B}(p_{i},p_{j}) $$

After separating the data, the new undirected weighted graph *G*^′^ is established by using the same method for the undirected weighted graph *G* and the weight calculation formula becomes

$$\begin{aligned} W^{\prime}\left\langle p_{i},p_{j} \right\rangle = f^{\prime}\left(b_{p_{i}},b_{p_{j}},A\right) - f^{\prime}(b_{p_{i}},b_{p_{j}},B) \end{aligned} $$

$$\begin{aligned} f^{\prime}(b_{p_{i}},b_{p_{j}},A) = \frac{ \left((1-\epsilon)^{2} + \epsilon^{2}\right) \times \widehat{N^{A}}(p_{i},p_{j}) + 2\epsilon \times (1-\epsilon) \times \widehat{N^{B}}(p_{i},p_{j})} { \widehat{N^{A}}(p_{i},p_{j}) + \widehat{N^{B}}(p_{i},p_{j}) } \end{aligned} $$

$$\begin{aligned} f^{\prime}(b_{p_{i}},b_{p_{j}},B) = \frac{ \left((1-\epsilon)^{2} + \epsilon^{2}\right) \times \widehat{N^{B}}(p_{i},p_{j}) + 2\epsilon \times (1-\epsilon) \times \widehat{N^{A}}(p_{i},p_{j})} { \widehat{N^{A}}(p_{i},p_{j}) + \widehat{N^{B}}(p_{i},p_{j}) } \end{aligned} $$

Where $\phantom {\dot {i}\!} \left | W^{\prime }\left \langle p_{i},p_{j} \right \rangle \right | > \delta $ represents an effective edge between 〈*p*_*i*_,*p*_*j*_〉 allelic sites. When adding it to the graph *G*^′^, the corresponding weight is $W^{\prime }\left \langle p_{i},p_{j} \right \rangle $. The default value of *δ* is 0.1. When the graph *G*^′^ is constructed, it can basically contain all the variation sites from the reference sequence. Then the maximum spanning tree *T* is extended to *T*^′^ according to the graph *G*^′^ following the steps in section Expanding local contigs.

In order to finally reconstruct the haplotype that contains the variants as many as possible, it is necessary to ensure that the extended tree *T*^′^ contains more variants. Lower coverage and higher threshold of coverage will cause some variation sites be left out. Thus, we automatically adjust to lower coverage threshold and edge weight threshold to ensure lower false-negative as much as possible. We adopt depth-first traversal of all vertexes in extended tree *T*^′^ and sort according to the relative positions in the order of reference sequence. After sorting, the state set of vertexes is a haplotype of the last sub-clone. Assuming that all variation sites are heterozygous, another haplotype of the last sub-clone is easily obtained. According to the linear evolution relationship among the sub-clones, the haplotypes of the remaining sub-clones are obtained by the following formula.

$$ h_{2i,j}=\left\{ \begin{array}{rcl} A, {h_{2(i+1),j} = A}\\ B, {h_{2(i+1),j} = B \ and \ sub(j) \leq i}\\ A, {h_{2(i+1),j} = B \ and \ sub(j) > i} \end{array} \right. $$

Where, *i* represents the label of sub-clone and *j* represents variation site number. *sub*(*j*) represents the sub-clone label of the *j*th variation site, *h*_2*i*,*j*_ represents the base state of the *j*th site from father chain on the *i*th sub-clone. By the above formula, we can find base state of the site from one haplotype corresponding to the paired haplotype.

$$ h_{2i,j}=\left\{ \begin{array}{rcl} h_{2i,j} \oplus 1, {sub(j) \leq i}\\ h_{2i,j}, {i < sub(j) \leq I-1} \end{array} \right. $$

**Fig. 4:**
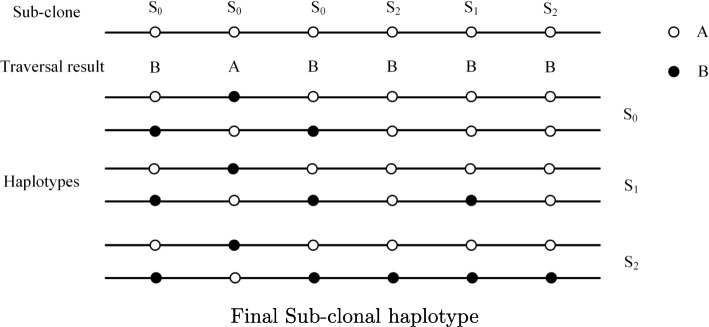
Final Sub-clonal haplotype

The construction process is shown in Fig. 4.

## Results

To generate simulation datasets, a chromosome is randomly selected from the human reference genome as a reference sequence. Simulating single point variation, germline mutation rate is set to 0.1%, and the somatic mutation rate is 1%. Consider the purity of the tumor sample: the founding clone *S*_0_ and two descendant sub-clone *S*_1_ and *S*_2_, the ratio of them is set to 3:5:2. Several parameters, such as coverage, the number of libraries, the length of read, have impact on the performance of *MixSubHap* algorithm, only one parameter value was changed for each experiment.

### Varying read length

Three libraries were set with different insert-sizes of 1000 bp, 1500 bp, and 2000 bp, respectively. Three sub-clonal mixing ratio is 3:5:2. A priori of sub-clone haplotype depends on the result of *SciClone*, whereas the accuracy of *SciClone* mainly depends on the library coverage and the sequence deviation of VAF. The library used for computing VAF is called base library. The coverage of the base library is set to be 100 ×, the coverage of other libraries is 50 ×, and the lengths of the paired-end reads are 100 bp, 150 bp, 200 bp, and 250 bp.

We have caculated the propotion of the clonal haplotypes we can recognize among all the variants, the accuracy rate of reconstruction, the longest length of fragment we can assemble, and the number of fragments assembled. The results are shown in Table 1. *MixSubHap* shows strong robustness, which can recognize over 90% clonal haplotypes, with the longest assembled fragments longer than 10 kbp under different read lengths. With the increase of read length, the recognition rate can be improved steadily.

**Table 1:** The accuracy of haplotype reconstruction under different read length

Read length	Recognition rate	Accuracy rate	Longest length	Fragments num
100bp	91.58%	60.50%	10068bp	4
150bp	91.84%	60.41%	10092bp	5
200bp	91.85%	60.50%	10091bp	5
250bp	92.15%	60.39%	10087bp	20

### Analysis on the influence of new library coverage

When selecting libraries and coverage, the base library and its coverage are generally determined firstly, from which the pair-end reads information covering at least two variants are extracted. Then we introduce new libraries until most of the variant sites are included. When the library is replaced, the library length is incremented by 500 bp by default. Influence of new library coverage on the accuracy of haplotype reconstruction and recognition rate in sub-clones are shown in Table 2. All the recognition rate is over 91%, and the longest assembled fragment is longer than 10077 bp. When the coverage of new library changes from 20 × to 100 ×, the change of recognition rate in sub-clone recognition is inconspicuous (less than 1%), indicating that a minor effect is caused by new libraries.

**Table 2:** The accuracy of haplotype reconstruction under different library coverage

Library num	Cov.(Base)	Cov.	Recognition	Accuracy	Longest length	Fragment num
3	100	20	91.61%	60.46%	10077bp	10
3	100	50	92.15%	60.39%	10087bp	20
3	100	80	92.00%	60.63%	10087bp	13
3	100	100	92.16%	60.35%	10091bp	19

### Analysis on the influence of the number of the libraries

We found that the coverage of the new library almost had no effect on the accuracy of the clonal haplotype reconstruction, so the next experiment was performed on the number of introduced libraries. The coverage of base library is set to 100 ×, and the other libraries are all set to 50 ×, which can reduce the cost of sequencing. The effect of the libraries number on the recognition accuracy of sub-clonal haplotypes is shown in the following Table 3.

**Table 3:** The accuracy of haplotype reconstruction under different number of libraries

Library num	Cov.(Base)	Cov.	Recognition	Accuracy	Longest length	Fragment num
1	100	50	89.00%	59.80%	9768bp	8
2	100	50	91.93%	60.10%	10097bp	12
3	100	50	92.15%	60.39%	10087bp	20
4	100	50	92.48%	60.28%	10091bp	32

As can be seen, the base library cannot recoginze the clonal haplotypes well. The recognition rate is less than 90%, the accuracy is less than 60%, and the longest assembled fragment is shorter than 10kbp. Meanwhile, we can see that the more libraries introduced to *MixSubHap*, the better the algorithm performs. Since the cost of sequencing sharply increases when the new library is added, we recommend two libraries with different insert-sizes to reconstruct the clonal haplotypes.

## Discussion

The proposed pipeline follows several constraints which imply the inheritance principle: Suppose that the micro evolution process of tumor tissue which satisfies the phylogenetic tree model [35, 45]. Assume that sub-clones in the tumor samples are in a linear evolution mode, the somatic mutations in the evolutionary process satisfying the two hit hypothesis, with the selective advantage, and the sites having the repair mechanism are not considered. In another word, one locus varies at most once in the process of evolution and the mutated site cannot be recovered. Thus, VAF is an important index to distinguish the various sub-clones, following certain inheritance principles in the process of sub-clone differentiation. For any variant site of *p*, VAF *V*_*p*_ is the number of reads supporting the mutation accounted for the proportion of the site’s sequenced depth, which can be statistically calculated from the sequencing reads data. We set the collection of sub-clone *S*_*i*_ ’s somatic mutation sites to be *M*_*i*_, among which *i*∈{0,1,…,*I*−1} and *I* is the total number of sub-clones. Clone *S*_0_ represents sub-clone with the largest common ancestor characteristics estimated from sequencing data, called the founding clone. The set of all the somatic mutations in sequencing samples is $ M = \bigcup _{i} M_{i} $. Let $G^{p}_{i}$ represents the genotype of site *p* on sub-clone *S*_*i*_, *S*_*j*_ represents the descendant clone of *S*_*i*_, where *i*<*j*. If *V*_*p*_=1, then *p*∈*M*_0_ and *p* is a homozygous mutation, while if *V*_*p*_=0.5, then *p*∈*M*_0_ and *p* is a heterozygous mutation. *V*_*p*_ and *V*_*q*_ are the VAF of variant *p* and *q*, if *V*_*p*_≤*V*_*q*_, then variant q belongs to the sub-clone which must be the descendant of the clone variant p belongs to. In addition, haplotype heterogeneity follows the inheritance principle that homozygous variation sites which are different from the reference sequence will not appear in clone. So $\sum _{k} V_{i,p,k} \leq 1$ and inheritance relationship in evolution process: If *V*_*i*,*p*,*k*_=1, then for all *i*^′^≥*i* we have $\phantom {\dot {i}\!} V_{i^{\prime },p,k} = 1 $, while if *V*_*i*,*p*,*k*_=0, then for all *i*^′^≤*i* we have $\phantom {\dot {i}\!} V_{i^{\prime },p,k} = 0 $. Among them, *V*_*i*,*p*,*k*_ indicates the VAF of variation site *p* is on the *k*th haplotype of sub-clone *S*_*i*_. The reconstruction of clonal haplotypes in tumor sample must satisfy the inheritance principle among sub-clones.

In addition, most of algorithms are based on the second generation sequencing data using linkage disequilibrium of haplotype. Different data input should also be considered [46]. the proposed pipeline consists of three components, which are assembling local VPEs, expanding local contigs and iteratively stripping clonal read-depth. These components are also prolongable to other types of sequencing data once the VPEs can be generated. Based on the second generation sequencing data, the VPEs are short relative to the sub-clonal haplotype, and the uncertainty is quite large: On the aspect of time complexity, if the tumor tissue contains *n* sites, there are 2^*n*^ haplotypes. *MixSubHap* processes VPEs that defines a proper partition length and connection, the short chains after partition can be accurately show sub-clonal haplotypes, and the time complexity is *O*((2*I*)!×*M*). Among them, *M* is the number of groups divided by the start position of the variation sites in VPE and *I* is the number of sub-clones. If the VPEs are extended, *M* may decrease significantly.

## Conclusions

The heterogeneity patterns on haplotypes are suggested to provide not only comprehensive information on tumor evolution and micro-environment, but valuable clinical implications as well. Most of the existing methods investigated the heterogeneity on genotype level, while the computational methods that facilitate the analyses on clonal haplotypes are in urgent needs. In this article, we presented *MixSubHap*, which is a computational pipeline for reconstructing clonal haplotypes. *MixSubHap* is able to identify about 90% on average of the preset clonal haplotypes under different simulation configurations. Especially, *MixSubHap* is robust when decreasing the mutation rates, in which cases the longest assembled contig could reach to 10kbps, while the accuracy of assigning a mutation to its haplotype still keeps more than 60% on average. According to the experimental results on the simulation datasets, we may conclude that the proposed pipeline is a practical tool working on cancer sequencing data. On the other hand, we also notice that two components of *MixSubHap* use the estimation of clonal structure to reduce the solution space. However, such estimation provided by the model-free methods yields more or less errors, especially when the VAFs are close among different sub-clones. This transferred errors could hurt the accuracy on reconstructing clonal haplotypes. Current version only considers point mutations, which will be further extended to structural variations and more complicated cases.

## Abbreviations

VAF: Variant allelic frequency
